# OA19 A case of severe refractory lupus enteritis and nephritis that remitted with Obinutuzumab

**DOI:** 10.1093/rap/rkac066.019

**Published:** 2022-09-28

**Authors:** Mithun Chakravorty, Philip Courtney

**Affiliations:** Royal Derby Hospital, Derby, United Kingdom; Nottingham University Hospitals, Nottingham, United Kingdom

## Abstract

**Introduction/Background:**

Systemic lupus erythematosus (SLE) is an autoimmune disease that can affect almost any organ system but gastrointestinal (GI) involvement is very rare. Obinutuzumab is a humanised type II anti-CD20 monoclonal antibody that is licensed to treat haematological malignancies. It has been used in a small number of SLE patients with secondary non-depletion non-response (2NDNR) to Rituximab and appears to be effective in lupus nephritis (LN) as outlined in the NOBILITY trial. We present a case of refractory lupus enteritis (LEn) and LN in a patient with 2NDNR to repeated Rituximab cycles that achieved remission with Obinutuzumab.

**Description/Method:**

A 19-year-old African woman was diagnosed with SLE in 2018 after presenting with polyarthritis without systemic involvement. Blood tests showed leucopenia, positive antinuclear antibody, anti-double-stranded DNA >200 IU/ml and hypocomplementaemia. Prednisolone and Methotrexate were commenced. However, one month later she presented to hospital with generalised abdominal pain, vomiting and diarrhoea. CT abdomen demonstrated significant small bowel wall thickening with mucosal hyperenhancement. Multidisciplinary team (MDT) input confirmed likely LEn. She was treated successfully with intravenous Methylprednisolone and Rituximab. Total parenteral nutrition was required for two weeks.

Unfortunately, four further hospital admissions followed over the next six months due to GI symptoms, transient acute kidney injury and moderate proteinuria. Small bowel oedema persisted on MRI, three months after the CT. Renal biopsy demonstrated class 2 LN. Intravenous methylprednisolone helped and further Rituximab was given but she had infusion reactions. Six months later she re-presented with abdominal pain, synovitis and had nephrotic range proteinuria. She was treated again for LEn after gastroenterology input. Further Rituximab was given (MabThera instead of Truxima) but reactions still occurred and her B cells were non-deplete. Repeat renal biopsy confirmed class 4 LN and intravenous Cyclophosphamide (Euro-Lupus regimen) was commenced. This was repeated seven months later due to persisting high disease activity and proteinuria despite a short trial of intravenous Belimumab. However, she was admitted to hospital with colitis on CT after completing the second course of Cyclophosphamide. MDT decision was to treat with Obinutuzumab. Since commencing treatment eighteen months ago, her SLE has dramatically improved and she feels well for the first time. SLEDAI-2K score is 2 (16 pre-treatment), renal function has recovered with mild proteinuria. Anti-double-stranded DNA has reduced from >200 to 52.9 IU/ml and complements normalised. She remains on Prednisolone 5mg daily and Tacrolimus and a third maintenance dose of Obinutuzumab is planned.

**Discussion/Results:**

LEn has been reported in only 0.2-5.8% of SLE cases. It is defined by the British Isles Lupus Assessment Group (BILAG 2004) by either vasculitis or inflammation of the small bowel supported by imaging or histology. Its pathogenesis is poorly understood but thought to be related to immune complex deposition and complement activation resulting in intestinal capillary leak and submucosal oedema. Symptoms are non-specific and a review by Janssens et al. (2013) suggested abdominal pain (97%), ascites (78%), nausea (49%), vomiting (42%) and diarrhoea (32%) were the most common symptoms in patients with LEn. They reported the ileum and jejunum were most commonly affected in LE (84% and 83% respectively), followed by colon (19%), duodenum (17%) and rectum (4%).

Abdominal CT is considered to be the first line investigation and ‘classic’ radiological findings are bowel wall oedema (“target sign”), engorgement/increased number of mesenteric vessels (“comb sign”) and increased attenuation of the mesenteric fat (Ha et al. 2000, and Kim et al. 2006). These were all demonstrated on our patient’s initial CT abdomen which showed engorgement of the mesenteric vessels from the proximal jejunum to the proximal ileum.

LEn seldom occurs in isolation and usually presents in the context of high disease activity and other systemic involvement. It is often responsive to glucocorticoids and bowel rest but further immunosuppression with Cyclophosphamide or Rituximab might be warranted in refractory cases as in our patient’s case.

Obinutuzumab has been shown to be safe, effective and steroid-sparing in renal and non-renal SLE patients with 2NDNR to Rituximab from a case series of 9 patients by Arnold et al. (2022). This applied to LN in our patient and we believe this to be the first case where refractory LEn was treated successfully with Obinutuzumab.

**Key learning points/Conclusion:**

Although very rare, have a high index of suspicion of LEn in SLE patients who present with GI symptoms and typical radiological findings.

LEn often occurs with other systemic manifestations including LN and it is important to assess for these at the time of diagnosis of LEn.

Obinutuzumab is an emerging treatment for severe SLE particularly 2NDNR to Rituximab and can improve both LN and LEn. We eagerly await results from the current REGENCY and ALLEGORY clinical trials which are assessing Obinutuzumab in renal and non-renal SLE patients respectively.

